# N-glycosylated SGK196 suppresses the metastasis of basal-like breast cancer cells

**DOI:** 10.1038/s41389-019-0188-1

**Published:** 2020-01-08

**Authors:** Ci Xu, Meichao Zhang, Lei Bian, Yanyan Li, Yuan Yao, Dong Li

**Affiliations:** Department of Radiation Oncology, Shanghai Ninth People’s Hospital, Shanghai Jiaotong University School of Medicine, Shanghai, China

**Keywords:** Breast cancer, Oncogenes, Metastasis

## Abstract

SGK196 is a protein *O*-mannose kinase involved in an indispensable phosphorylation step during laminin-binding glycan synthesis on alpha-dystroglycan (α-DG). However, the function of SGK196 in cancer diseases remains elusive. In the current study, we demonstrated that SGK196 is primarily modified by N-glycosylation in breast cancer (BC) cells. Furthermore, gain and loss-of-function studies showed that N-glycosylated SGK196 suppresses cell migration, invasion, and metastasis in BC, particularly in the basal-like breast cancer (BLBC) type. In addition, we found that SGK196 N-glycosylation performs the regulatory function through the PI3K/AKT/GSK3β signaling pathway. Collectively, our results show that N-glycosylated SGK196 plays suppression roles in BLBC metastases, therefore providing new insights into SGK196 function in BC.

## Introduction

As one of the leading causes of mortality in females, breast cancer (BC) has posed a great threat to their health^1,2^. One of the significant advances in cancer research in the past few years is the molecular categorization of cancer based on gene expression profiles^3,4^. Transcriptomic analyses of human breast tumors have led to classification of four major intrinsic molecular subtypes that include luminal A, luminal B, human epidermal growth factor receptor 2-enriched (HER2^+^) and basal-like breast cancer (BLBC)^5^. BC’s molecular categorization is strongly associated with patients’ survival outcomes, among which BLBC has the worst prognosis^6^. BLBC consists of about 10–15% of all breast cancer types, characterized by high proliferation and invasion, high expression of basal cytokeratins (CKs) and low expression of the estrogen receptor (ER), progesterone receptor (PR) and HER2 [ref. ^7–10^]. The lack of these hormone receptors has made chemotherapy the unique choice for tumor treatment, although resistance is always observed^11,12^. Thus, it is urgently needed to further dissect the molecular mechanism of BLBC so that personalized therapy can be developed for patients.

The *SGK196* gene (synonym *POMK*) is located on chromosome 8p11 and encodes the protein O-mannose kinase, which is composed of 350-amino acid residues and predicted to have a molecular weight of 40.15 kDa. As a type II transmembrane protein, SGK196 consists of a cytoplasmic tail, one transmembrane helix, and a luminal domain. It is universally expressed in skeletal muscle, brain, heart and kidney tissues. Because of the lack of functional motifs of protein kinases including Asp-Phe-Gly (DFG), His-Arg-Asp (HRD) and conserved Lys residues in the primary structure, SGK196 had been previously regarded as a “pseudokinase”^13^. However, it was recently shown that SGK196 functions as a glycosylation-specific kinase. By using ATP as a donor substrate, SGK196 is involved in the phosphorylation of M3 glycan Mannose on the C-6 position within the M3 O-glycan of alpha-dystroglycan (α-DG)^14,15^. α-DG is a peripheral membrane protein, serving as a receptor for various extracellular matrix components, such as laminin, agrin, perlecan^16^. Extensive glycosylation of α-DG is essential for its binding to extracellular matrix ligands^17–19^. Mutations of *SGK196* can influence the biosynthesis of functional α-DG, causing a spectrum of congenital and limb-girdle muscular dystrophies^20–23^.

As one of the most common post-translational modifications, glycosylation is involved in multiple cellular events including migration, proliferation, adhesion, and apoptosis^24,25^. Glycosylation is accomplished through collaboration between cellular glycosyltransferases and glycosidase, which add or trim glycans on asparagine (N-linked glycan) or serine/threonine (O-linked glycan) residues in polypeptides in the endoplasmic reticulum and the Golgi apparatus^26–28^. In addition, increasing evidence has revealed the vital role of glycosylation alterations in malignant transformation and metastasis. Alterations in protein glycosylation can result in the impairment of cell-cell adhesion, enhanced migration, lymphohematogenous invasion, and activation of intracellular oncogenic pathways.

To date, the role of SGK196 in cancer disease has not yet been reported, while the potential mechanism underlying its function is even less clear. In this study, we found that SGK196 can be modified by N-glycosylation at position Asn67, Asn165, Asn220, and Asn235. Gain and loss-of-function studies showed that N-glycosylated SGK196 inhibits cell migration, invasion, and metastasis in vitro and in vivo of BLBC cells. In addition, we have also unveiled the molecular biological mechanism by which SGK196 N-glycosylation modulates BLBC migration and invasion.

## Results

### The potential post-translational modification of SGK196 may involve in the development of BLBC

**Fig. 1:**
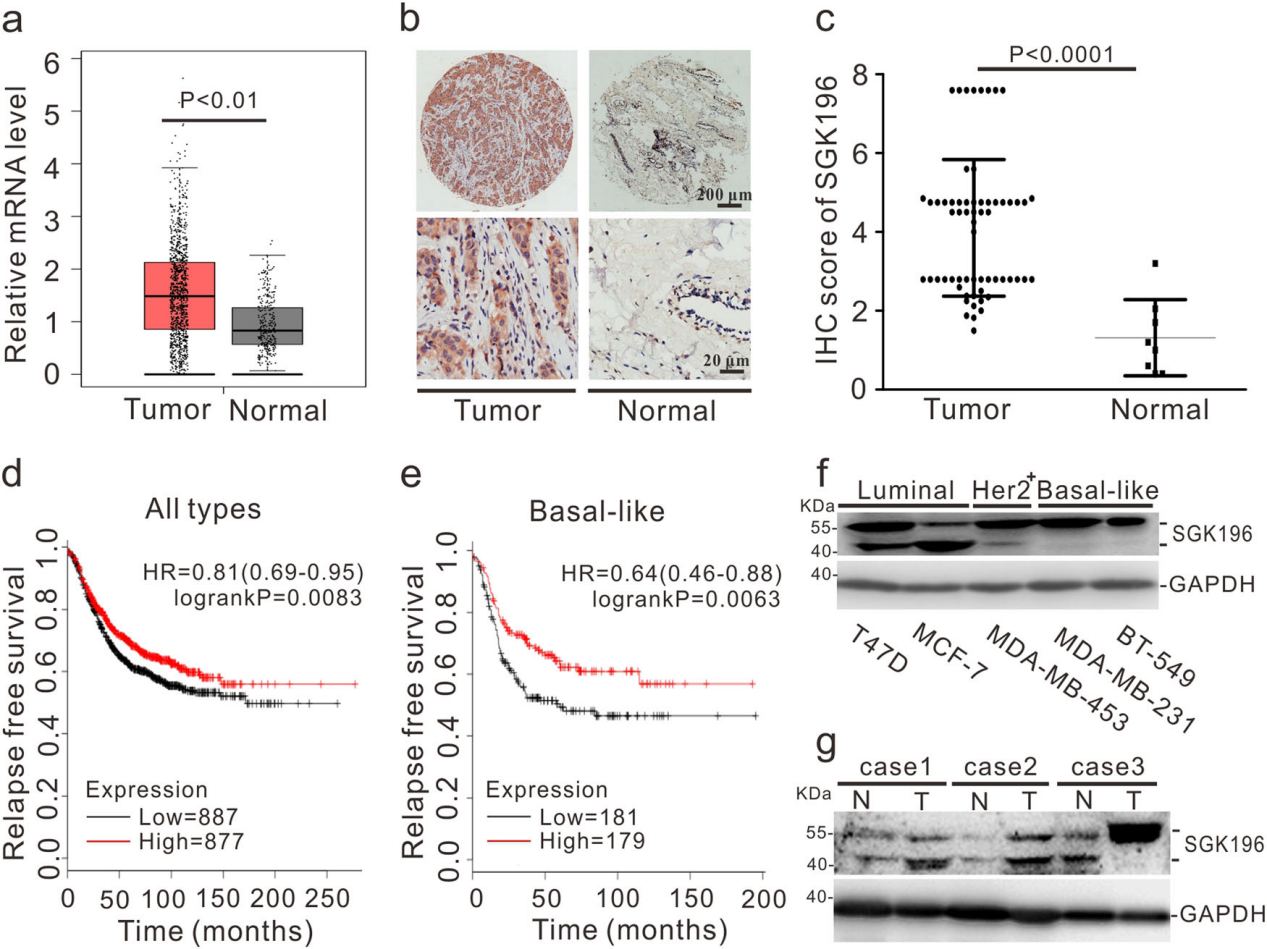
The potential post-translational modification of SGK196 may involve in the development of BLBC. **a** GEPIA database shows the mRNA level of *SGK196* in BC tissue and in normal breast tissues (num (*T*) = 1085; num (*N*) = 291). **b** Representative images of SGK196 expression in tissue microarray chip. **c** Scatter plots depicting the expression of SGK196 in BC tissues (*n* = 63) and normal adjacent breast tissues (*n* = 8). **d**, **e** RFS curves were plotted for all BC patients (**d**) and RFS curves were plotted for basal-like type patients (**e**). Data was analyzed using Kaplan-Meier Plotter. Patients with high expression are indicated in red line, and patients with low expression in black line. HR means hazard ratio. The *P*-value was calculated by a log-rank test. **f** Immunoblotting analysis of SGK196 expression in different subtypes BC cell lines. **g** Immunoblotting analysis of SGK196 expression in 3 pairs of BC tissues and adjacent normal tissues. (N: adjacent normal breast tissues; T: BC tissues; case1 and case 2 belong to luminal type, while case 3 belongs to basal-like type).

To determine *SGK196* mRNA expression in BC, GEPIA (http://gepia.cancer-pku.cn/) and ONCOMINE (http://www.oncomine.org/) microarray databases were applied for analysis. The relative mRNA level of *SGK196* was significantly higher in human BC tissues than that in normal breast tissues (Fig. 1a), while no significant difference was observed among the four subtypes (Fig. S1A). To detect the protein level of SGK196 during tumorigenesis, the tissue microarray chip including 63 cases of BC tissues and 8 cases of normal adjacent breast tissues was examined by immunohistochemistry and the data showed that SGK196 protein expression (brown staining area) is significantly increased in BC tissues comparing to the normal adjacent breast tissues (*P* < 0.0001) (Fig. 1b, c; Fig. S1B). But intriguingly, the higher mRNA level of *SGK196* is correlated with better RFS in BC patients (*P* = 0.0083) according to analyses by log-rank tests in the Kaplan-Meier survival plots, especially in subtypes of BLBC patients (*P* = 0.0063) (Fig. 1d, e). However, there was no significant statistical difference between *SGK196* mRNA level and RFS of BC patients in other BC subtypes (Fig. S1C–E). In order to further illustrate the characteristics of SGK196 protein, Western blot assay was conducted in various subtypes of breast cancer cell lines (T47D, MCF-7, MDA-MB-453, MDA-MB-231, and BT-549) as well as breast cancer tissues and their adjacent normal tissues. To our interest, SGK196 protein bands shifted differently in BC cell lines (Fig. 1f). Same phenomenon was also observed in breast cancer tissues and adjacent normal ones (Fig. 1g; Fig. S1F). Particularly, SGK196 protein bands were detected predominantly at 55 kDa in basal-like breast cancer cell lines (MDA-MB-231, BT-549) and in breast cancer tissues of the basal-like type (Fig. 1f, g). Herein, we hypothesize that the potential post-translational modification of SGK196 instead of the total mRNA or protein level might play a critical role in BLBC and be probably also in correlation with the patients’ survival.

### SGK196 is modified via N-linked glycosylation in BC cells

**Fig. 2:**
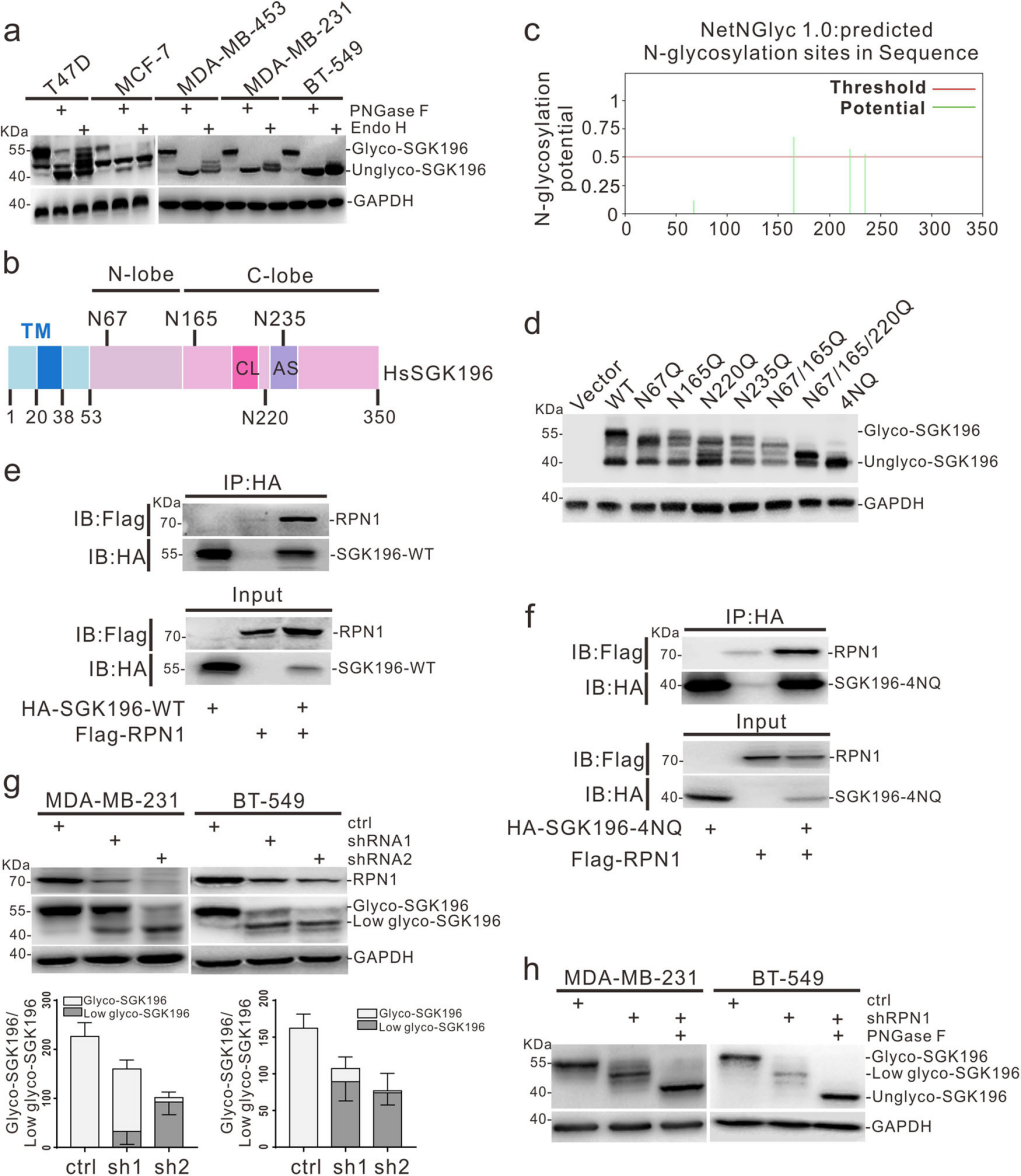
SGK196 is modified via N-linked glycosylation in BC cells and partially regulated by RPN1. **a** Cell lysates from different subtypes BC cell lines were treated with PNGase F or Endo H, and then were used for Western blotting analysis of SGK196 protein. **b** Schematic illustration of human SGK196 protein and its potential N-glycosylation sites. **c** Predicted N-glycosylation sites of human SGK196 by NetNGlyc1.0 Server. **d** Cell lysates from HEK293 cells transiently transfected with HA-tagged SGK196-WT or -mutants were collected and subjected to Western blotting analysis. 4NQ represents N67/165/220/235Q mutation. **e**, **f** Co-immunoprecipitation analysis in HEK293 cells revealing that RPN1 interacts with both SGK196-WT (**e**) and SGK196-4NQ (**f**). **g** Western blotting analysis of glycosylation status of SGK196 in MDA-MB-231 cells and BT-549 cells infected with control shRNA and RPN1 shRNA and the relative ratio of the upper to the lower form of SGK196 was quantified when RPN1 expression was diminished in MDA-MB-231 cells and BT-549 cells. **h** Western blotting analysis of glycosylation status of SGK196 in MDA-MB-231 cells and BT-549 cells infected with RPN1 shRNA and control shRNA; molecular weight shifts are compared with or without PNGase F digestion treatment.

It was shown previously that there are potential N-glycosylation sites in human SGK196 protein^14^. To test this, we obtained lysates from different subtypes of BC cell lines and digested them with PNGase F or Endo H enzymes, which can efficiently and specifically remove N-linked oligosaccharides from glycoproteins^29^. Western blotting analysis showed that N-glycosylation of SGK196 was seen in all showed BC cell lines, while other forms of glycosylated SGK196 might also exist in T47D and MCF-7 cells, as illustrated by the results from PNGase F or Endo H treatments (Fig. 2a). To identify the site(s) of N-glycosylation, we performed bioinformatics analysis, which revealed four potential N-glycosylation sites in human SGK196 protein (Fig. 2b, c). To determine the sites at the functional level, we generated a series of SGK196 mutants (N to Q), expressed them in HEK-293T cells, and analyzed using western blot assay. We found that the SGK196-4NQ mutant was completely unable to be glycosylated (Fig. 2d). The PNGase F or Endo H treatment assay also indicated that SGK196-4NQ mutant was totally deglycosylated (Fig. S2A). Further, we overexpressed SGK196-WT and SGK196-4NQ in MDA-MB-231 and BT549 respectively (Fig. S2B). The NQ mutations did not affect the stability (Fig. S2C) or the subcellular localization of SGK196 protein in both MDA-MB-231 and BT-549 cells (Fig. S2D). Thus, SGK196 is mainly N-glycosylated in human BC cells.

### SGK196 is regulated partially by RPN1

To address how SGK196 N-glycosylation might be regulated, we performed SGK196 immunoprecipitation (IP) followed by mass spectrometry to identify potential SGK196-associated protein(s), which enabled us to find RPN1 (ribophorin I) in the SGK196 precipitates (Fig. S3A and Table 2). RPN1 reportedly acts as an important part of N-oligosaccharyl transferase (OST) complex^30^. The interaction between SGK196 and RPN1 was further confirmed with Western blotting analysis (Fig. 2e). Furthermore, the 4NQ mutation did not prevent the binding of SGK196 to RPN1 (Fig. 2f).

Since SGK196 could be N-glycosylated, it was possible that RPN1 might be able to regulate the glycosylation level of SGK196. We depleted RPN1 in MDA-MB-231 and BT-549 cells using specific shRNAs and found the molecular weight of SGK196 was greatly diminished (Fig. 2g). To confirm if the decrease of SGK196 molecular weight was due to deglycosylation, we treated cell lysate with PNGase F to remove N-glycan chains, which further decreased the molecular weight of SGK196 compared with the lysates from cells with RPN1 depletion (Fig. 2h). It was worth noting that the total SGK196 protein level was also reduced upon RPN1 depletion (Fig. 2g), and the reduction might be due to the decrease in *SGK196* mRNA levels after RPN1 depletion (Fig. S3B, C). Finally, to ask whether SGK196 impacted the expression of RPN1, we depleted endogenous SGK196 expression using two specific shRNAs against SGK196 or overexpressed SGK196 in MDA-MB-231 and BT-549 cells. We found that SGK196 depletion or overexpression caused little effect on RPN1 mRNA and protein levels (Fig. S3D, E). Together, these results suggested that RPN1 regulates, at least partially, the N-glycosylation of SGK196.

### SGK196 inhibits the migration and invasion of BLBC cells

**Fig. 3:**
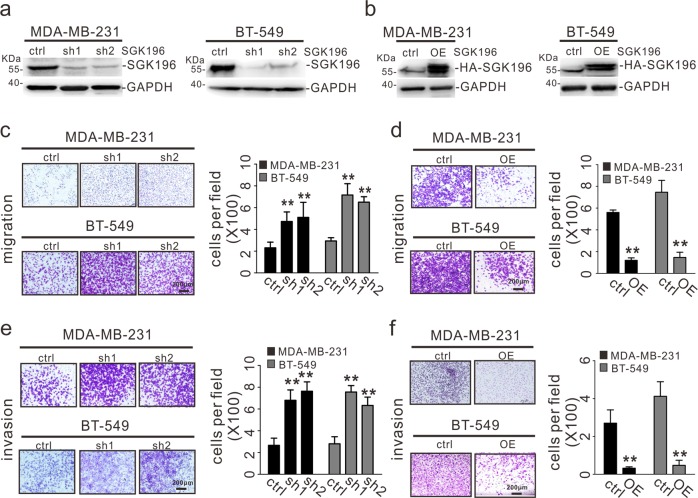
SGK196 inhibits the migration and invasion of BLBC cells. **a** MDA-MB-231 and BT-549 cells were infected with control shRNA and two different SGK196 shRNAs. After antibiotic selection, knockdown of SGK196 was validated by Western blotting analysis. **b** MDA-MB-231 and BT-549 cells stably expressing HA-SGK196 were validated by western blotting analysis. **c**–**f** The resultant stable cell lines with SGK196 knockdown and overexpression were subjected to migration assays (**c**, **d**) and invasion assays (**e**, **f**). The data are presented as the mean ± S.D, **p < 0.01.

Having shown that SGK196 might have a role in the development of BLBC, we sought to investigate the cellular function and the underlying molecular mechanism. To do so, we established two BLBC cell lines (MDA-MB-231 and BT-549)^31^ with stable SGK196 depletion or expression using retroviral or lentiviral infection and subsequently lysed the cells for Western blotting analysis, which showed successful SGK196 depletion or ectopic expression (Fig. 3a, b). SGK196 depletion in both MDA-MB-231 and BT-549 cells significantly increased cell migration and invasion (Fig. 3c, e). In contrast, MDA-MB-231 and BT-549 cells with SGK196 ectopic expression exhibited markedly reduced migration and invasive ability compared with the control without overexpression (Fig. 3d, f). The function of SGK196 in migration and invasion appeared specific. SGK196 depletion/overexpression exerted no effects on cell proliferation or colony formation in either of the cell lines (Fig. S4A–D).

### SGK196 N-glycosylation is required for repression of metastasis

**Fig. 4:**
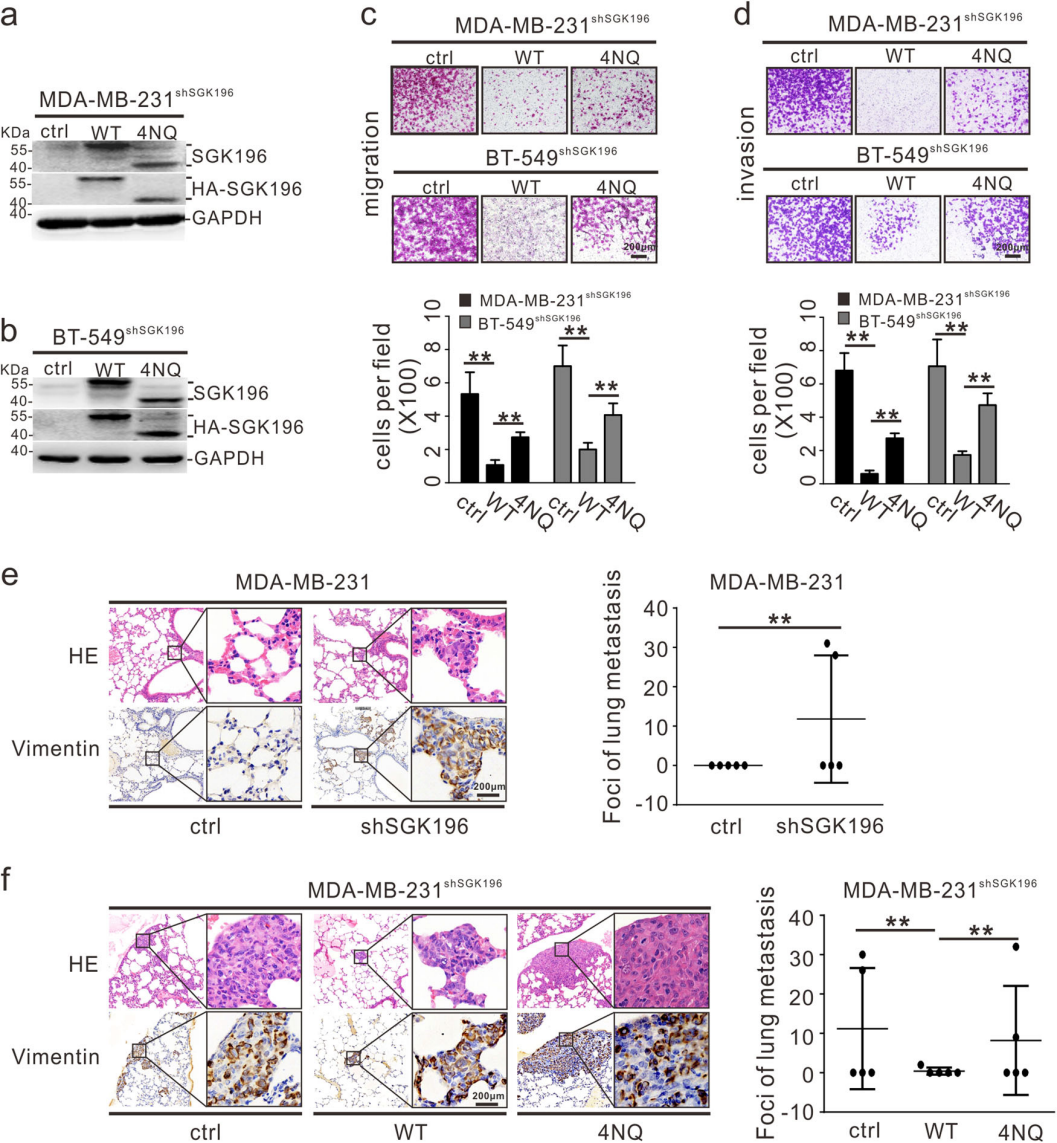
SGK196 N-glycosylation is required for repression of metastasis. **a**, **b** Endogenous SGK196 was knocked down in MDA-MB-231 and BT-549 cells by shRNAs, and HA-SGK196-WT and HA-SGK196-4NQ were re-expressed thereafter in MDA-MB-231^shSGK196^ and BT-549 ^shSGK196^ cells by use of lentiviral infection. Expression of endogenous and exogenous SGK196 protein in these cell lines was validated by Western blotting analysis. **c**, **d** The stable cell lines were subjected to migration assays (**c**) and invasion assays (**d**). The data are presented as the mean ± S.D, **p < 0.01. **e** MDA-MB-231 cells were infected with retroviral vectors containing control shRNA or SGK196 shRNA. The stable cell lines were injected into nude mice via tail vein, and lungs were harvested 6 weeks after injection. Representative images of HE-staining and human vimentin staining of lung tissues are shown. The data are presented as the mean ± S.D, **p < 0.01. **f** Endogenous SGK196 was knocked down in MDA-MB-231 cells by shRNAs, and HA-SGK196-WT and HA-SGK196-4NQ were re-expressed thereafter in MDA-MB-231^shSGK196^cells by lentiviral infection. The stable cell lines were injected into nude mice via tail vein, and lungs were harvested 6 weeks after injection. Representative images of HE staining and human vimentin staining of lung tissues are shown. The data are presented as the mean ± S.D, **p < 0.01.

We next assessed the role of SGK196 N-glycosylation in the regulation of metastasis by constructing cell lines MDA-MB-231^shSGK196^ and BT-549^shSGK196^, which had endogenous SGK196 depleted and could be readily resupplied with exogenously expressed SGK196-WT and SGK196-4NQ (Fig. 4a, b). This system allowed us to rule out potential artifacts created by exogenous SGK196 overexpression. Experiments with several functional assays demonstrated that re-supply/re-expression of SGK196-WT significantly inhibited the migration and invasion abilities of cells compared with MDA-MB-231 ^shSGK196^ or BT-549 ^shSGK196^ cells (Fig. 4c, d). In contrast, re-expression of SGK196-4NQ mutant only partially rescued the effects caused by SGK196 depletion (Fig. 4c, d). Expectedly, re-expression of SGK196-WT or SGK196-4NQ had little effect on cell proliferation and colony formation (Fig. S4E–G).

To confirm the findings from the in vitro experiments, we evaluated the role of SGK196 N-glycosylation in BLBC metastasis in vivo. For lung metastasis assay, we injected MDA-MB-231 cells into nude mice via the tail vein, and metastatic lung nodules were confirmed *via* hematoxylin and eosin (H&E) staining. In addition, the lung tissues were also stained for human vimentin to specifically identify MDA-MB-231 cells. We discovered many more lung metastasis nodules in the MDA-MB-231-shSGK196 group than those in the MDA-MB-231-shctrl group (Fig. 4e). In contrast, re-supply of WT-SGK196 in MDA-MB-231^shSGK196^ cell line significantly decreased the number of lung metastasis nodules when compared with the empty vector. Instead, re-supply of the 4NQ-SGK196 mutant, unlike WT-SGK196, failed to decrease lung metastasis nodules (Fig. 4f). Thus, loss of N-Glycosylated SGK196 modestly increased the lung metastatic ability of MDA-MB-231 cells in vivo.

### N-glycosylated SGK196 suppresses metastasis by inhibiting the PI3K/AKT/GSK3β signaling pathway

**Fig. 5:**
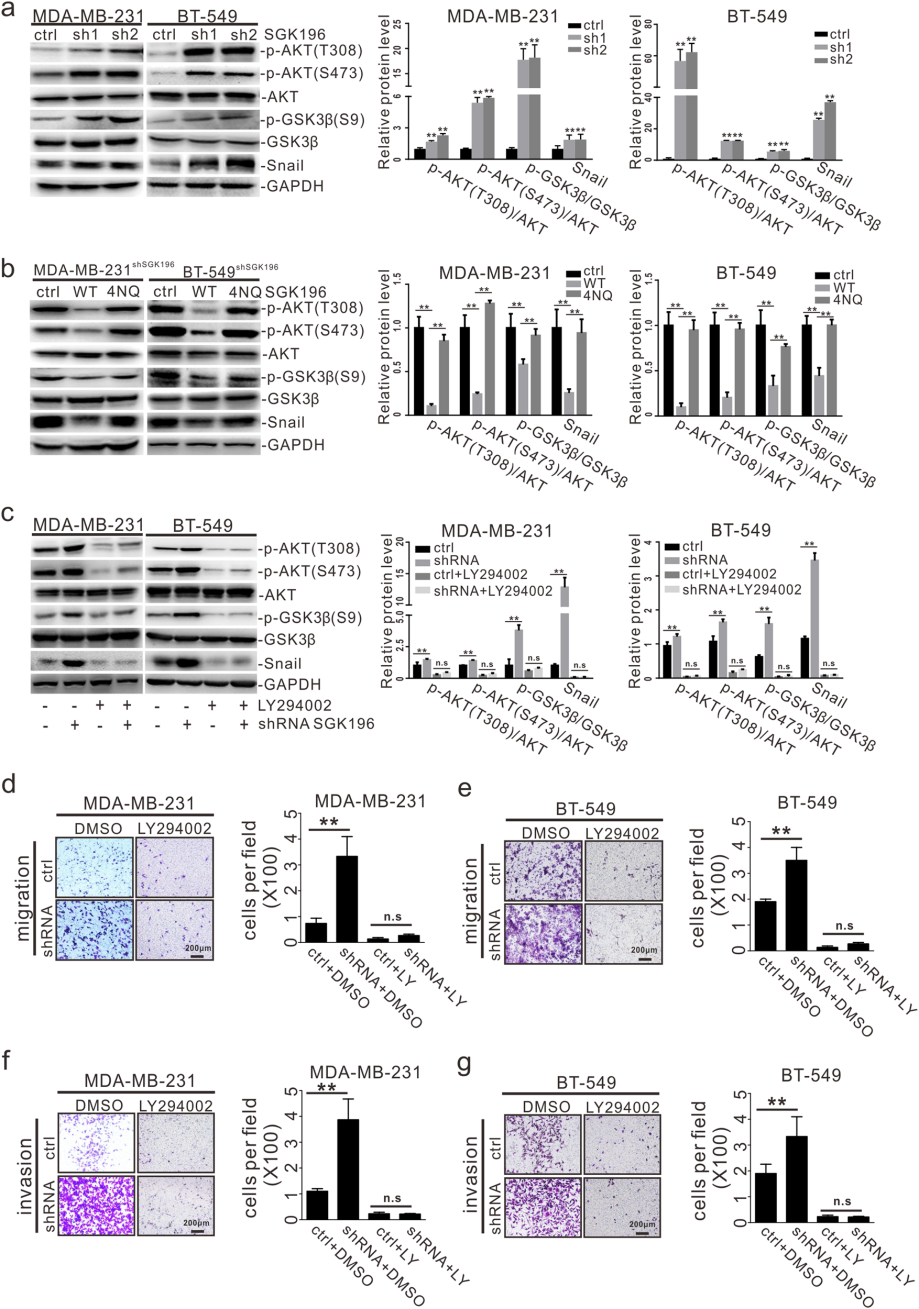
N-glycosylated SGK196 suppresses metastasis by inhibiting the PI3K/AKT /GSK3β signaling pathway. **a** Western blotting analysis showing that phosphorylation of AKT and GSK3β and Snail expression were elevated after knockdown of SGK196 in MDA-MB-231 and BT-549 cells. Densitometric analysis was performed for AKT and GSK3β phospholyation and for the levels of Snail. **b** Western blotting analysis showing that the increase of p-AKT, p-GSK3β, and Snail caused by knockdown of SGK196 was reversed by re-expression of SGK196-WT, but not SGK196-4NQ, densitometric analysis was performed for AKT and GSK3β phospholyation and for the levels of Snail. **c** Western blotting analysis showing that the increase of p-AKT, p-GSK3β and Snail expression caused by knockdown of SGK196 was prevented by LY294002, an inhibitor of PI3K. Densitometric analysis was performed for AKT and GSK3β phospholyation and for the levels of Snail. **d**–**g** The MDA-MB-231 and BT-549 cells with depleted SGK196 were treated with 10 μM LY294002 and subjected to migration (**d**, **e**) and invasion assays (**f**, **g**). The data are presented as the mean ± S.D, **p < 0.01.

To explore the mechanism through which SGK196 regulates BLBC metastasis, we first explored the relationship between SGK196 and its known substrate α-DG. Western blot assay showed whether knockdown or overexpression of WT SGK196/4NQ SGK196 had little impact on the expression of α-DG in MDA-MB-231 and BT-549 cells (Fig. S5A, B). Then, we assessed the impact of SGK196 depletion on signaling pathways known to be involved in tumor metastasis. It was demonstrated that SGK196 depletion caused the level of phosphorylated AKT (p-AKT) to elevate in both MDA-MB-231 and BT-549 cells by Western blotting analysis (Fig. 5a), while re-expression of SGK196-WT restored the p-AKT level caused by SGK196 depletion (Fig. 5b). In contrast, re-expression of SGK196-4NQ failed to reverse the level of p-AKT (Fig. 5b). It is well known that AKT regulates breast cancer metastasis by inhibiting GSK-3β activity and subsequently leading to Snail stabilization. We therefore hypothesized that SGK196 might inhibit the BLBC metastasis through the PI3K/AKT/GSK-3β/Snail pathway. SGK196 depletion induced activation of AKT and GSK-3β, followed by elevated Snail protein levels (Fig. 5a). In contrast, re-expression of SGK196-WT, but not SGK196-4NQ, significantly suppressed the activation of AKT and GSK-3β (Fig. 5b). We also treated the MDA-MB-231-shSGK196/BT-549-shSGK196 cells with LY294002 (a specific inhibitor against PI3K), which effectively decreased the levels of p-AKT, p-GSK-3β and Snail caused by SGK196 depletion (Fig. 5c). Moreover, the increase in cell migration and invasion induced by SGK196 depletion was substantially inhibited by the addition of LY294002 (Fig. 5d–g). Thus, SGK196 N-glycosylation is required for SGK196 suppression of PI3K/AKT/GSK-3β signaling pathway and its anti-metastasis function in BLBC.

## Discussion

Through gene-expression profiling, researchers have identified BLBC as a distinct BC subtype characterized by highly aggressive behaviors, refraction to treatment and poor prognosis^32–34^. Without targeted therapies, patients are susceptible to a high risk of relapse and high rate of death when they are treated with traditional chemotherapy^35,36^.

In this work, we specifically investigated the biological function of either SGK196 or N-glycosylated-SGK196 in BLBC, due to a higher expression of *SGK196* mRNA being significantly in correlation with better RFS (Fig. 1e). It was suggested that SGK196 might be an oncogene because of its higher expression level in breast cancer tissues. However, SGK196 appears to execute an anti-invasion function in vitro (Fig. 3d, f) and anti-metastasis function in vivo (Fig. 4f). Intriguingly, varied forms of protein band of SGK196 were observed in both human breast cancer cell lines and breast cancer tissues according to the Western blot detection (Fig. 1f, g; Fig. S1F). With this, the glycosylation of SGK196 raised our concern and was identified by a series of biological analysis.

As a post-translational modification, N-glycosylation has an important influence on many physiological processes, including the occurrence and development of cancer. Our site-directed mutagenesis studies provide the first evidence that there are four N-linked glycosylation sites in human SGK196 protein, and N-glycosylated SGK196 is the only glycosylation form of SGK196 existing in BLBC cell lines (Fig. 2a–d). Meanwhile, we discovered an N-oligosaccharyl transferase RPN1, which is thought to be responsible for catalyzing the transfer of a high mannose oligosaccharide to nascent polypeptide chains, partially regulated the glycosylation of SGK196 in MDA-MB-231 and BT-549 cells (Fig. 2g)^37,38^. Thus, there might be other types of N-glycan chains (complex or hybrid N-glycans), which can be catalyzed independently by RPN1 and can be conjugating on SGK196 in MDA-MB-231 and BT-549 cells, while various numbers of high mannose oligosaccharides conjugating on SGK196 exist in there two cell lines, despite of the fact that both of the cell lines belong to the BLBC type. In addition, when we depleted RPN1, both the mRNA and protein level of SGK196 was reduced (Fig. 2g; Fig. S3B, C). We speculate that RPN1 also regulates the N-glycosylation of certain membrane proteins, which in turn influence downstream signaling pathway(s), leading to the activation/inactivation of undefined transcription factors or transcriptional regulators. These transcription factors or transcriptional regulators might affect mRNA expression of SGK196.

Accumulating evidence has shown that N-linked glycosylation is involved in a large number of biological processes, including protein folding, cell-cell interactions, protein trafficking and signal transduction^39–41^. In our study, we also attempted to understand the role of SGK196 N-glycosylation in metastasis of BLBC cells. Based upon our current findings from in vitro and in vivo studies, SGK196 N-glycosylation is capable of repressing metastasis of BLBC cells (Fig. 4c, d, f).

To dissect the mechanism by which SGK196 N-glycosylation regulates metastasis of BLBC, we determined the impact of SGK196 depletion on several key signaling pathways known to be involved in metastasis. The depletion of SGK196 in MDA-MB-231 or BT-549 cells leads to marked activation of the AKT-GSK3β-Snail signaling axis that reportedly contributes to the acceleration of the metastasis process in BC or other types of cancer (Fig. 5a)^42–44^. In keeping with the notion, deglycosylation of SGK196 induces a similar activation of AKT-GSK3β and enhances Snail protein levels (Fig. 5b). In addition, knockdown or overexpression of WT SGK196 /4NQ SGK196 had little impact on the expression of α-DG (Fig. S5A, B). Together, these results strongly suggest that N-glycosylated SGK196 plays a role in suppressing the metastasis of BLBC cells mainly through the AKT-GSK3β pathway and that the interaction between SGK196 and α-DG may differ in cancer cells and muscular/neuron cells. In cancer cells, SGK196 may interact with other substrates, which in turn may affect the AKT-GSK3β signaling way. Further studies are needed to elucidate the detailed underlying mechanism.

In summary, we find that high levels of N-glycosylation of SGK196, which is mediated partially by RPN1, may have a critical role in suppression of metastasis of BLBC. At the mechanistic level, N-glycosylated SGK196 exerts its effect on metastasis mainly by inhibiting the AKT-GSK3β signaling pathway. Our findings provide new insights into the biological function of SGK196 and may offer a potential new approach for BC treatment.

## Materials and methods

### Cell culture and cell lines

Human breast cancer cell lines (MDA-MB-231, BT-549, MCF-7, T47D, MDA-MB-453) and human embryonic kidney 293T (HEK-293T) cell lines were purchased from Cell Bank of Type Culture Collection of Chinese Academy of Sciences (Shanghai, China). All of the cells were cultured in a modified medium supplemented with 10% fetal bovine serum (FBS, 1600044, Gibco, MA), 100 U/mL penicillin, 100 mg/mL streptomycin (15140122, Gibco), and 1% GlutaMAX (35050061, Gibco). MDA-MB-231, MDA-MB-453, HEK-293T cells were specifically cultured in high-glucose DMEM (SH30243.01, Hyclone, UT), BT-549 cells in RPMI 1640 (SH30809.01, Hyclone), MCF-7 cells in DMEM/F12 (SH30023.01, Hyclone). The number of passages of all the cell lines did not exceed five. Cell transfection was performed using Hiff TransTM Liposomal Transfection Reagent (40802ES02, YEASEN, Shanghai, China). Cell infection was mediated by the use of polybrene (40804ES76, YEASEN).

### Antibodies and reagents

Antibodies used in this study include Anti-SGK196 (ab57908, Abcam, Cambridge, UK); Anti-GSK3β (A0480, Abclonal, Wuhan, China), Anti-p-GSK3β-S9 (AP0039, Abclonal); Anti-RPN1 (sc48367, Santa Cruz, Dallas, TX); Anti-p-AKT-Ser473 (#4058, Cell Signaling Technology, Danvers, MA), Anti-p-AKT-Thr308 (#13038, Cell Signaling Technology), Anti-AKT(pan) (#2920, Cell Signaling Technology), Anti-Snail (#3879, Cell Signaling Technology); Anti-HA (H6908, Sigma-ALDRICH, MO), Anti-Flag (F1804, Sigma-ALDRICH), Vimentin (sc-66001,Santa Cruz), α-dystroglycan (VIA4) (sc-53986, Santa Cruz) and Anti-GAPDH (HC301-01, TRANSGEN BIOTECH, Beijing, Shanghai). All antibodies for Western blotting were used at dilution 1:1000. For Immunofluorescence assays, Anti-HA (H6908, Sigma-ALDRICH) was used at dilution 1:100. Other reagents include DMEM medium (11965-084, Gibco), RPMI 1640 medium (11875-085, Gibco), Cell Counting Kit reagents (40203ES60, YEASEN), puromycin (P8230, Solarbio, Beijing, China), LY294002 (L9908, Sigma-ALDRICH), CHX (5087390001, Sigma-ALDRICH), Protease inhibitor Cocktail (EDTA-Free, 100x in DMSO, 20124ES03, YEASEN), Endo H (# P0702S, New England Biolabs, Ipswich, MA), and PNGase F (# P0705S, New England Biolabs).

### Tumor specimens and tissue microarray

All human breast tumor tissues and their corresponding adjacent normal breast tissues were obtained from surgically resected samples from BC patients in Shanghai Ninth People’s Hospital affiliated to Shanghai Jiao Tong University School of Medicine. All patients involved in this study did not receive any radiotherapy or chemotherapy previously and were consented to participate in the study and publish the findings. This study was approved by Committee of Experiments Research at Shanghai Jiao Tong University School of Medicine. Human breast cancer tissue microarray chip (SHANGHAI OUTDO BIOTECH CO. LTD, China) includes 71 samples (63 human breast cancer samples and 8 normal breast tissue samples).

### Immunohistochemical and H&E staining

Xylene and different grades of alcohol were used to deparaffinize and rehydrate the tissue microarray chip before soaking with 3% H_2_O_2_ for 15 min. Sodium citrate buffer (PH 6.0) was used to retrieve antigen for 2 min, followed by incubation with anti-SGK196 (1:300 dilution) at 4 °C overnight. The tissue microarray chip was then incubated with secondary antibody for 20 min at 37 °C after washes with PBS. Slides were stained with DAB and counterstained with hematoxylin. The expression level of SGK196 was quantified based on the immunohistochemical scoring criterion as follows: 0, no expression; 1–2, weak expression, <10%; 3–5, moderate expression, between 10–50%; 6–8, high expression, >50%. Immunohistochemical scoring was carried out independently by three pathologists in a blind manner. The lungs of nude mice were fixed in 10% formalin, embedded in paraffin, and sectioned serially. Afterward, the tissue sections were deparaffinized, stained with H&E and carried out immunohistochemical staining of human vimentin to confirm the lung metastases.

### Plasmids, site-directed mutagenesis, transfection, and retrovirus/lentivirus infection

For shRNA-mediated knockdown of SGK196, LMP-puro-shRNA containing retroviruses were used. The target sequences of SGK196 shRNAs were shRNA-1: 5′-GTCTTGGATACACTTAGA-3′, and shRNA-2: 5′-AGTTACAGCATTCTACTCT-3′. For shRNA-mediated knockdown of RPN1, pLKO.1-puro-shRNA containing lentiviruses were used. The target sequences of RPN1 shRNAs were shRNA-1: 5′-GCCTTTCTCACGCTATGATTA-3′ and shRNA-2: 5′-GTGAAGCTTGCCTCTCGAAAT-3′. For encoding protein with different tags, various vectors were used. Briefly, pCMV-Tag-2b vectors for FLAG-RPN1, pEF5HA vectors for HA-SGK196, pCD513B-1 lentivirus vectors for HA-SGK196. pCD513B-1 lentivirus vectors for rescuing SGK196 (i.e. knockdown of endogenous SGK196 with SGK196 shRNA targeting 3-UTR sequence and overexpression of HA-tagged SGK196). Mutations of SGK196 were generated with mu-primers using KOD enzyme (KOD-201, TOYOBO, Japan), and Exnase® II enzyme (C215-01/02, Vazyme, Nanjing, China) was used for DNA recombination. Forward and reverse primer sequences for each N-to-Q mutation are shown in Table 1. Mutations were confirmed using automatic DNA sequencing.

### Cell proliferation assay

Cell Counting Kit-8 (CCK8) was used according to the manufacturer’s recommended instructions. Briefly, cells were cultured in 96-well plates, 90 μL of complete culture medium and 10 μL of CCK-8 reagent were mixed and added to each well. The absorbance was measured at 450 nm after 2 h of incubation at 37 °C.

### Colony formation assay

MDA-MB-231 (0.5 × 10^3^) and BT-549 (0.5 × 10^3^) cells were seeded in 6-well plates, and medium was changed every 3 days. After two weeks, cell colonies were stained by 0.01% crystal violet, and the number of colonies with at least 50 cells was counted under a microscope at ×4 magnification.

### Extraction of total RNA and Real-time PCR assay

Total RNA was extracted with TRIzol reagent (15596-026, Invitrogen, Carlsbad, CA) according to the manufacturer’s protocol. The reverse transcription of RNA samples was performed with PrimeScript RT reagent Kit with gDNA Eraser (RR047A, TAKARA, Shiga, Japan). Afterward, Real-time PCR reaction was performed using SYBR Green Master Mix Reagent (11201ES03, YEASEN) and run in the Roche Light Cycler 480 Real-Time PCR detector. The primers used in Real-time PCR are SGK196-F: 5′-ACCCTCTAGGTTCCTTGAGT-3′ and SGK196-R: 5′-GTTGGAGTCGCACATGACC-3′; GAPDH-F: 5′-ATGAGGTCCACCACCCTGTT-3′ and GAPDH-R: 5′-CTCAAGGGCATCCTGGGCTA-3′. Results were expressed at a relative mRNA level and analyzed using the comparative threshold cycle (2^−ΔΔCT^) method with GAPDH as the reference gene.

### Western blotting

Cells or tissues were lysed in lysis buffer, and a BCA Protein Assay Kit (Beyotime Biotechnology Co, Jiangsu, China) was used to measure protein concentrations. Equal amounts of total protein were loaded, ran on 10% SDS-polyacrylamide gel, and transferred to PVDF membranes (Millipore, Billerica, MA). The membranes were blocked with Tris-buffered saline containing 5% nonfat milk for 2 h and probed with primary antibodies of target proteins and GAPDH at 4 °C overnight followed by incubation with horseradish peroxidase-linked secondary antibodies (1:5000) for 1 h at room temperature. Signals were detected with ECL reagents (ThermoFisher, Waltham, MA).

### Co-immunoprecipitation

Cells were washed three times with phosphate-buffer saline, harvested, and lysed in co-immunoprecipitation buffer (50 mM Tris-HCl, pH 7.4, 150 mM NaCl, 1% NP-40, 0.1% SDS, 1 mM EDTA and a complete protease inhibitor cocktail). Cell lysates (1 mg) were incubated with 8 μL of PureProteom^TM^ Protein A/G Mix Magnetic Beads (Millipore USA) and appropriate antibodies overnight. The beads were then washed three times and boiled to isolate the protein. Further analysis was carried out with sodium dodecyl sulfate polyacrylamide gel electrophoresis separation and subsequently analyzed with Western blotting or mass spectrometry. Mass spectrometry was conducted and analyzed by HOOGEN BIOTECH (Shanghai, China).

### Immunofluorescence and confocal microscopy

Cells were allowed to adhere to pre-coated glass coverslips overnight. They were fixed the next day in 4% paraformaldehyde and blocked with 5% BSA in PBS for 1 h. Primary antibody-treated cells were washed with PBS and then incubated with Alexa Fluor®555 donkey anti-rabbit secondary antibodies (ab150074, abcam) at a 1:500 dilution in PBS for 1 h. Cell nuclei were dyed with DAPI (C1002, Beyotime) for 5 min. Finally, the cells were mounted using glycerol and observed using the Lecia Tcs SP8 confocal microscope.

### In vitro migration and invasion assays

Cell migration and invasion assays were carried out using Transwell chambers (Corning, NY) with or without Matrigel (BD Biosciences, MD) coating. Briefly, cells (5 × 10^4^/well) were plated in serum-free medium onto the upper compartment of the transwell chamber. Medium containing 10% FBS was added to the lower compartment as a chemical attractant. After incubation for 12–36 h, cells that had migrated or invaded into the lower surface of the filters were fixed by paraformaldehyde, stained with crystal violet, photographed and counted in 5 random fields with a Nikon ECLIPSE Ts2R microscope.

### Animal xenograft

Five-week-old female nude mice (five mice per group) were injected *via* tail vein with 1 × 10^6^ cells in 100 μL PBS. Mice were observed for every three days and sacrificed before natural death occurred. Lungs were collected and fixed in 4% buffered formalin solution for further study. All animal studies were performed using protocols approved by the Ethics Committee of Experiments Research at Shanghai Jiao Tong University School of Medicine.

### Statistical analysis

All experiments were performed at least three times. Values are expressed as mean ± S.D. (standard deviation). The significance of the difference between any two samples was analyzed with *T*-test using Graph Pad Prism 6; values of *p* < 0.05 were considered statistically significant.

## Supplementary information

Supporting Materials

Table S1

Table S2

## Data Availability

All data generated or analyzed during this study are included in this published article and its supplementary information files.
