# Supplementary material for: N-glycosylated SGK196 suppresses the metastasis of basal-like breast cancer cells
Source: Oncogenesis. 2020 Jan 8;9(1):4. doi: 10.1038/s41389-019-0188-1 (PMC6949223; doi:10.1038/s41389-019-0188-1)
Supplement: Supplementary file 1 — Supporting Materials [file 41389_2019_188_MOESM1_ESM.docx]

**Supporting Figures**

**
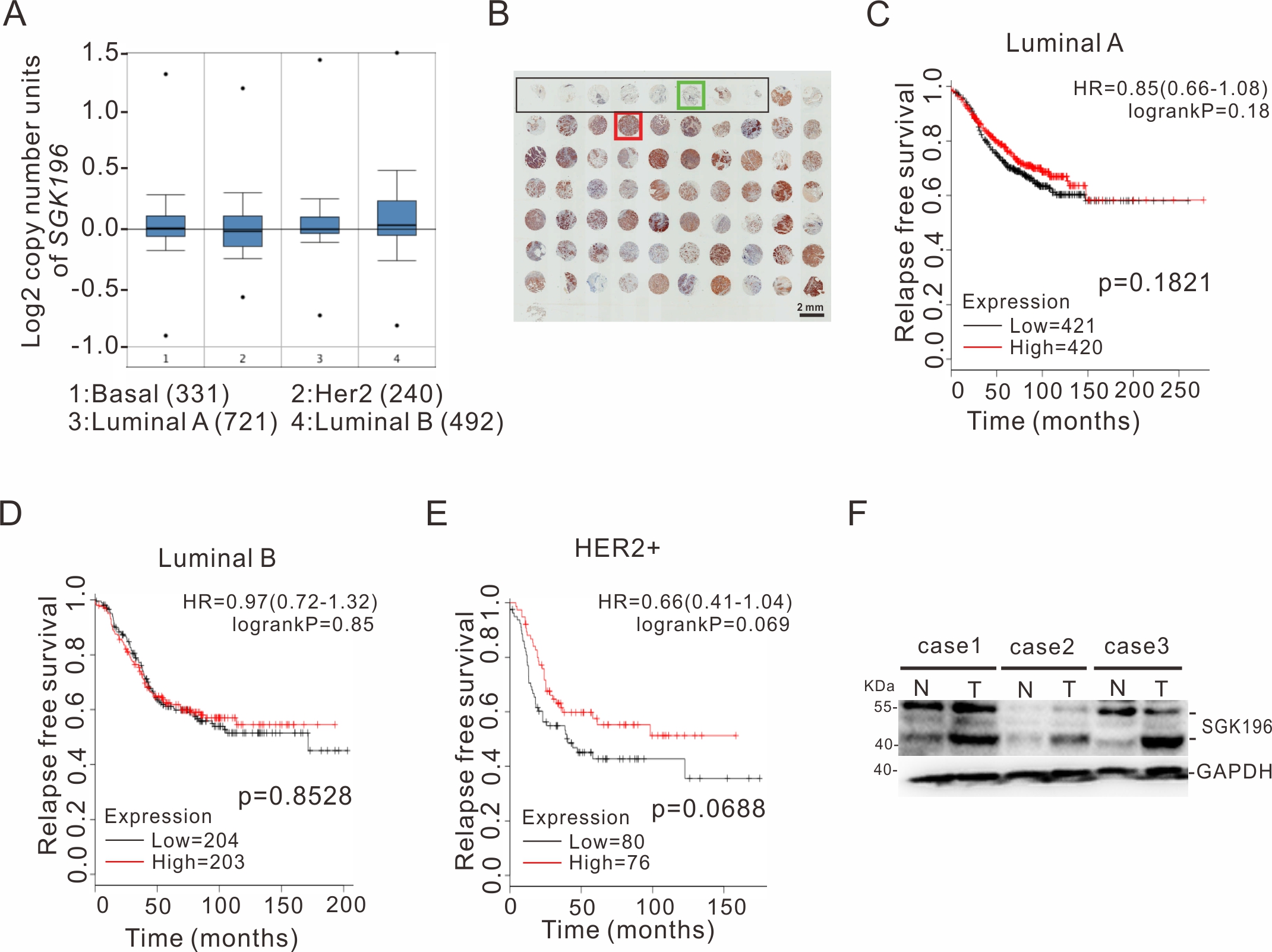
Fig. S1.** (A) ONCOMINE database shows the expression of SGK196 mRNA levels in different BC subtypes (num (Basal) =331; num (HER2) =240; num (Luminal A) =721; num (Lumnial B) =492). (B) Images of SGK196 protein staining in TMA chip containing 63 cases of breast cancer tissues and 8 cases of normal adjacent breast tissues (indicated in black frame) as revealed by IHC. The representative images of normal adjacent breast tissue (indicated in green frame) and breast cancer tissue (in red frame) are shown in Figure 1C. (C-E) RFS curves were plotted for Luminal A type patients (C), Luminal B type patients (D), and Her2+ type patients (E). (F) Immunoblotting analysis of SGK196 expression in 3 pairs of BC tissues and adjacent normal tissues. (N: adjacent normal breast tissues; T: BC tissues; three cases all belong to luminal type).

**
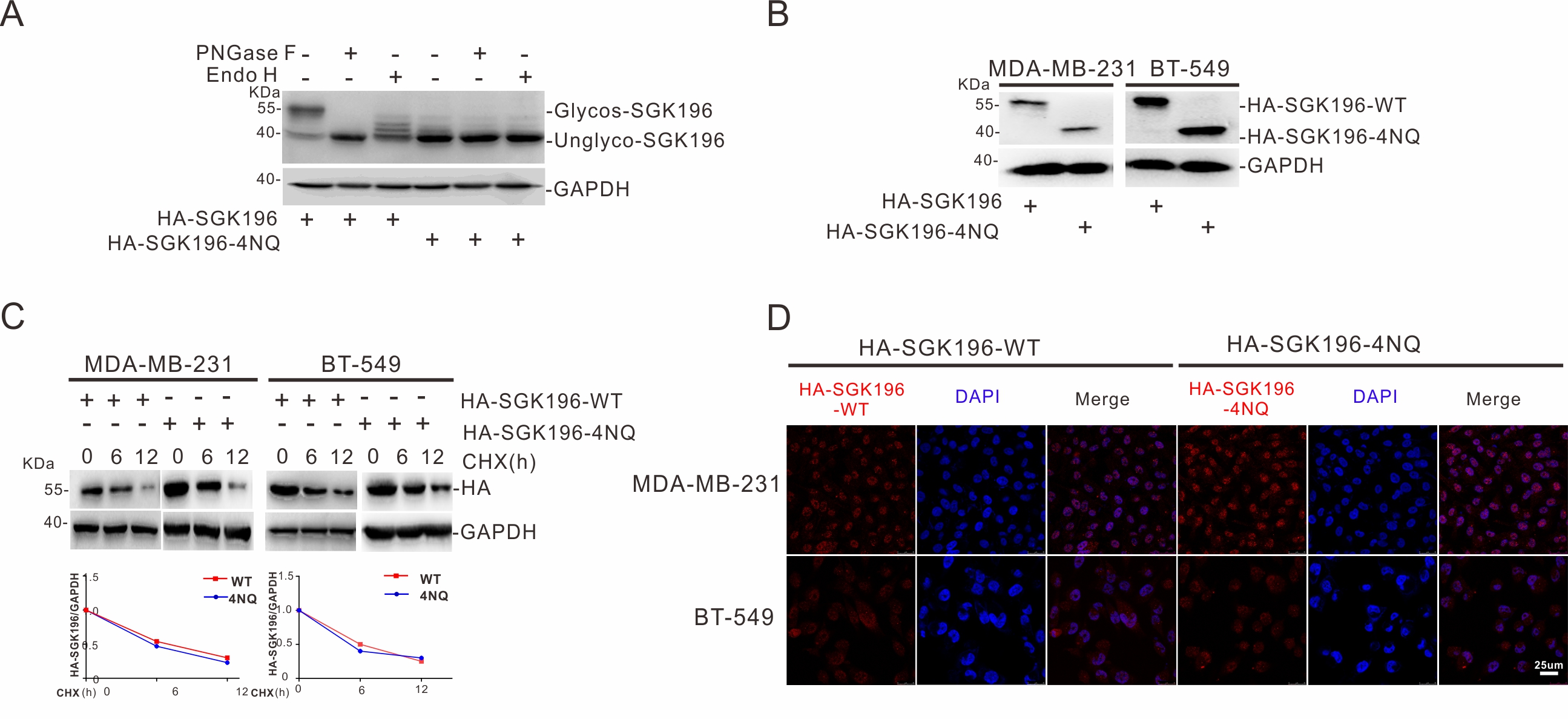
Fig. S2.** (A) HEK-293T cells were transiently transfected with HA-SGK196-WT or HA-tagged SGK196-4NQ for 48 h, and cell lysates were treated with PNGase F or Endo H for immunoblotting analysis. (B) MDA-MB-231 or BT-549 cells stably expressing HA-tagged SGK196-WT or HA-tagged SGK196-4NQ were generated using lentiviral infection and analyzed with immunoblotting. (C) Cells stably expressing HA-tagged SGK196-WT and HA-tagged SGK196-4NQ were treated with 100 μg/ml of cycloheximide (CHX) for the indicated time and then subjected to immunoblotting analysis with the HA antibody. (D) Cells stably expressing HA-tagged SGK196-WT and HA-tagged SGK196-4NQ were subjected to immunofluorescence staining with an anti-HA antibody. Cell nuclei were counterstained with DAPI.

**
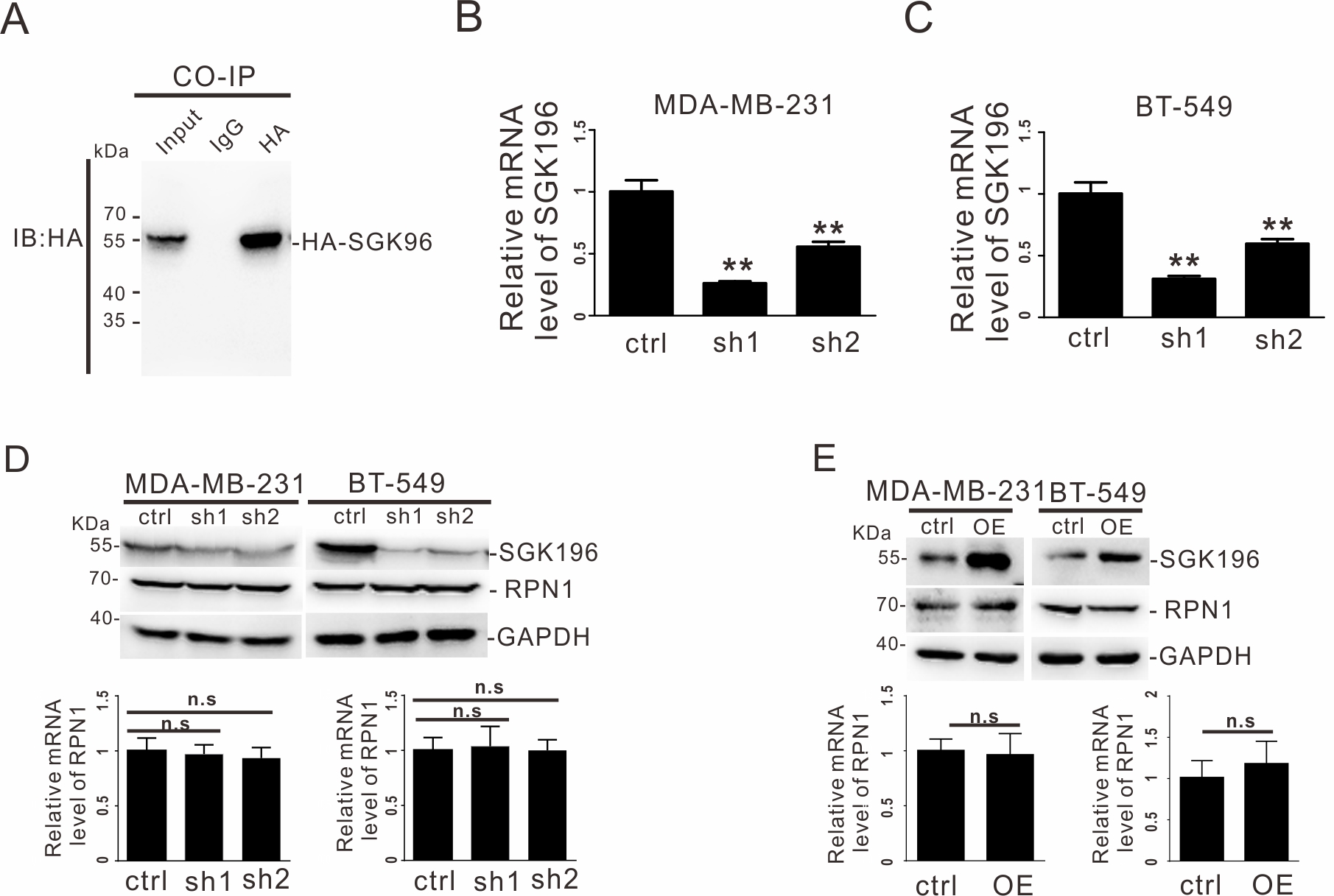
Fig. S3.** (A) Lysates from MDA-MB-231 cells after immunoprecipitation was used for mass spectrometry analysis. (B, C) Real-time PCR analysis of SGK196 mRNA in MDA-MB-231 cells with either control shRNA or RPN1 shRNA (B) and in BT-549 cells with either control shRNA or RPN1 shRNA (C). (D) Western blotting and Real-time PCR analysis of RPN1 protein in MDA-MB-231 cells and in BT-549 cells containing either control shRNA or SGK196 shRNA. (E) Western blotting analysis and Real-time PCR of RPN1 protein in MDA-MB-231 cells and in BT-549 cells containing either control vector or HA-tagged SGK196-WT.

**
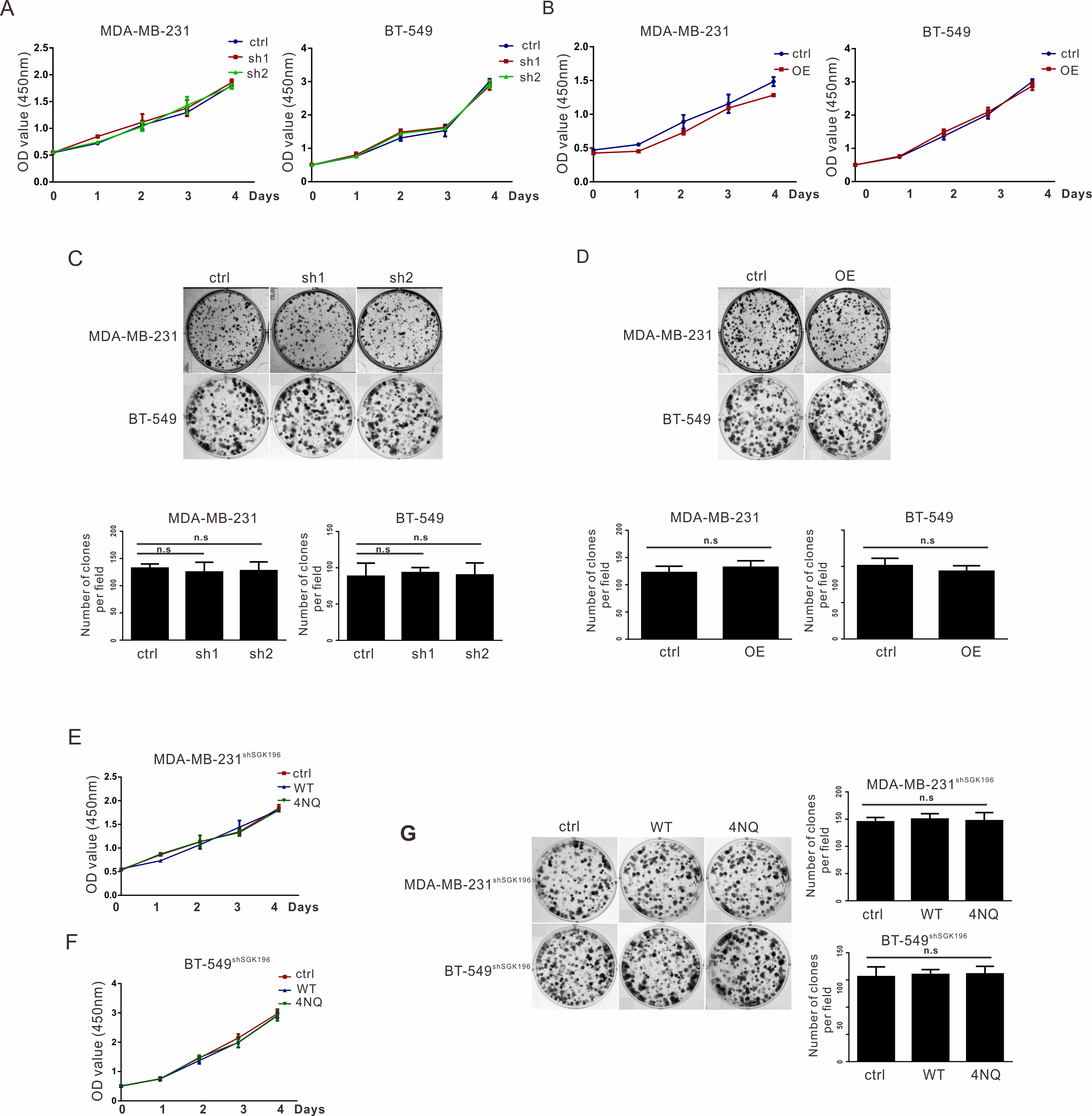
Fig. S4.** (A, B) The stable cell lines with depleted or overexpressed SGK196 were subjected to cell viability analysis using CCK8 assays. (C, D) The stable cell lines with depleted or overexpressed SGK196 were subjected to colony formation assays and the number of colonies was quantified. (E, F) Endogenous SGK196 was knocked down in MDA-MB-231 and BT-549 cells by shRNAs targeting SGK196. Afterwards, HA-tagged SGK196-WT or HA-tagged SGK196-4NQ was re-expressed in MDA-MB-231^shSGK196^ and BT-549 ^shSGK196^ cells by using lentiviral infection. The stable cell lines were subjected to cell viability analysis using CCK8 assays. (G) The indicated stable cell lines were subjected to colony formation assays, and the number of colonies was quantified.

**
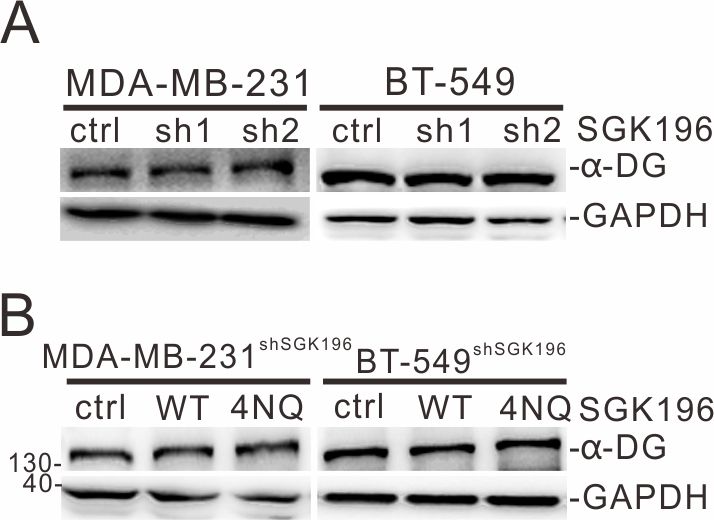
**

**Fig. S5.** (A) Western blotting analysis of α-DG in MDA-MB-231 cells and BT-549 containing with either control shRNA or SGK196 shRNA. (B) Western blotting analysis of α-DG in MDA-MB-231 ^shSGK196^ or BT-549 ^shSGK196^ cells with re-expression of either SGK196-WT or SGK196-4NQ.
