# Supplementary material for: N-glycosylated SGK196 suppresses the metastasis of basal-like breast cancer cells
Source: Oncogenesis. 2020 Jan 8;9(1):4. doi: 10.1038/s41389-019-0188-1 (PMC6949223; doi:10.1038/s41389-019-0188-1)
Supplement: Supplementary file 2 — Table S1 [file 41389_2019_188_MOESM2_ESM.docx]

**Table S1.** Primer sequences for generating N-to-Q mutations

| Name | Sequence 5’-3’ |
| --- | --- |
| N67Q-F | 5’-AAACAATGCTCACCTTGGCTGTCCTGCGAGGAG- 3’ |
| N67Q-R | 5’-AGGTGAGCATTGTTTCATCTGTCCTATCCTGAA -3’ |
| N165Q-F | 5’-CTACAACTTTCAAAGTACCAAAATGTGAAC ACG- 3’ |
| N165Q-R | 5’-CTTTGAAAGTTGTAGTGTTTCTTCCAGGTT ACT -3’ |
| N220Q-F | 5’-AGCCAATTCAGCATTTTGGCAAATGACTTG GAC -3’ |
| N220Q-R | 5’-AATGCTGAATTGGCTTGTTAGCAGATACTGGGA -3’ |
| N235Q-F | 5’-GTGCAACACAGCTCCGGGATGCTGGTGAAGTGC-3’ |
| N235Q-R | 5’-GGAGCTGTGTTGCACCAGGGGTAAGGCGTCCAA- 3’ |
