# Supplementary material for: N-glycosylated SGK196 suppresses the metastasis of basal-like breast cancer cells
Source: Oncogenesis. 2020 Jan 8;9(1):4. doi: 10.1038/s41389-019-0188-1 (PMC6949223; doi:10.1038/s41389-019-0188-1)
Supplement: Supplementary file 3 — Table S2 [file 41389_2019_188_MOESM3_ESM.docx]

**Table S2.** List of potential proteins interacting with SGK196

| NO. | Mol.Weight(kDa) | Names and Offical Symbol | Relative Abundance(%) |
| --- | --- | --- | --- |
| 1 | 49.921 | Dolichyl-diphosphooligosaccharide-protien glycosyltransferase subunit 1, RPN1 | 37.3 |
| 2 | 20.107 | Peroxiredoxin-2, PRDX2 | 20.71 |
| 3 | 112.89 | Sodium/potassium-transporting ATPase subunit alpha-1, ATP1A1 | 19.79 |
| 4 | 25.211 | 40S ribosomal protein S2, RPS2 | 16.04 |
| 5 | 163.29 | Alpha-2 macroglobulin, A2M | 9.34 |
| 6 | 34.733 | Serine/threonine-protein phosphatase I regulatory subunit 10, PPP1RS10 | 4.41 |
| 7 | 10.905 | 40S ribosomal protein S30, RPS30 | 3.51 |
| 8 | 36.408 | OUT domain-containing protein 4, OTUD4 | 2.74 |
| 9 | 19.779 | Heterogeneous nuclear ribonucleoprotein L, HNRNPL | 2.57 |
| 10 | 35.73 | BTB/POZ domain-containing protein KCTD12 | 0.98 |
